# The Importance of Patient Expectations: A Mixed-Methods Study of U.S. Psychiatrists

**DOI:** 10.3389/fpsyt.2021.781494

**Published:** 2021-12-03

**Authors:** Maayan N. Rosenfield, Michael H. Bernstein

**Affiliations:** Center for Alcohol and Addiction Studies, School of Public Health, Brown University, Providence, RI, United States

**Keywords:** placebo, expectancies, psychiatry, non-specific factors, hope

## Abstract

**Objective:** To examine how psychiatrists think about and modulate non-specific factors (e.g., hope, expectations) in clinical practice.

**Methods:** U.S. psychiatrists were recruited for two studies assessing attitudes and behaviors related to non-specific factors. Study 1 entailed remote qualitative focus groups (*k* = 7) with *n* = 26 participants (36.0% female). Study 2 was a quantitative survey with *n* = 346 respondents (34.0% female) designed to assess the generalizability of focus group findings.

**Results:** Four themes were identified in Study 1 that were used to inform the survey (Study 2): (1) Expectations (2) Hope, (3) Placebo Effect, and (4) Aesthetic Features. Nearly all surveyed psychiatrists (92.2%) considered patient expectations at least “most of the time” when interacting with a patient. Focus groups revealed that psychiatrists often attempt to balance optimism and realism to improve outcomes. A majority of survey respondents believed office design and physician attire could at least somewhat influence expectations (72.5 and 77.3%, respectively) and even outcomes (51.5 and 58.7%, respectively). Focus group psychiatrists described how physical features may be used as therapeutic tools.

**Conclusions:** Psychiatrists are highly mindful of patient expectations. Although there is variability in the perceived importance of expectations, hope, the placebo effect, and aesthetic features, many utilize these factors in clinical practice.

## Introduction

While medical research typically focuses on specific treatment factors, such as the chemical ingredients in pills or specific psychotherapeutic techniques, a growing body of research indicates that non-specific factors also contribute to patient outcomes (1–3). As inflation-adjusted healthcare costs have nearly doubled since the start of the 21st century (4), it is critical to investigate these non-specific factors as low-cost ways to improve patient outcomes. Non-specific factors refer to general aspects of treatment common across therapeutic modalities, such as trust, rituals, hope, or expectations (5). For example, better physician communication (e.g., more eye contact) and increased empathy have a small but statistically significant effect on both subjective and objective patient outcomes such as weight loss, health-related quality of life, and re-consultation rate (6). High expectations can trigger dopamine and endogenous opioid release, which have a healing effect (7, 8).

Non-specific factors contribute to patient outcomes in psychiatry (9). In a study of patients with depression, the physician effect accounted for nearly 3 times more variance in depression score than medication effect (10). Furthermore, patients with depression receiving placebos experience, on average, 82% of the improvement of patients taking antidepressants (11), in large part due to the expectation that the pill will help them (12, 13). The converse has also been demonstrated; an expectation that one *might* receive a placebo rather than active medication can reduce efficacy of an antidepressant (14). Non-specific factors can also contribute to symptom improvement in a variety of psychiatric conditions besides depression (15). Given non-specific factors' importance to psychiatry, an important step in maximizing their utilization is assessing psychiatrist attitudes.

A limited number of studies demonstrate that while psychiatrists have favorable attitudes toward non-specific factors, they underestimate their contribution to treatment effectiveness. Among 87 German physicians (40.2% psychiatrists, 25.3% neurologists, 24.1% general practitioners), patient expectations were considered the second most important contributor to antidepressant effectiveness, after pharmacological effects (16). In a survey of 79 psychiatrists and trainees at a U.S. academic hospital, 96% endorsed the statement, “enhancing therapeutic components that contribute to placebo responsivity may be a clinically appropriate way of improving clinical outcomes” (17, p. 4). While 76% of Canadian psychiatrists surveyed (*n* = 257) reported ever prescribing an impure placebo [e.g., vitamins or subtherapeutic doses of medications; see (17)], only 20% said they had prescribed placebos in clinical practice (18), suggesting many do not consider impure placebos to actually be placebos. While psychiatrists recognize that the placebo effect can ameliorate symptoms, they may underestimate its magnitude. Psychiatrists believe 26% (19) to 40% (16) of antidepressant effectiveness can be attributed to the placebo effect, lower than academic estimates (11).

Past research has indicated that expectancies play a large role in psychiatric treatment and that psychiatrists generally have favorable attitudes toward the placebo effect. However, to our knowledge, no prior work has investigated if and how psychiatrists utilize non-specific factors in conjunction with therapy and medication to maximize treatment effectiveness in clinical practice. We investigate these questions in a mixed methods study of U.S. psychiatrists.

## Methods

### Qualitative Study

#### Recruitment

Names and emails were obtained from a commercially available listing of 10,949 psychiatrists across the U.S. 5,105 randomly selected psychiatrists were sent up to three recruitment emails inviting them to participate in a virtual focus group on “how psychiatrists utilize expectations” and “how factors outside of what is commonly thought of as treatment (such as therapeutic alliance or aesthetic features of an office) can affect patient outcomes.” Participants received no compensation. The study was approved by the Brown University IRB.

#### Participants

Forty practicing or recently retired psychiatrists signed up and 26 attended one of 7 focus groups (size: 2–6, *M* = 3.7). Participants completed residency 4–47 years ago (*M* = 30.3, *SD* = 12.5) and 36.0% were female. Participants hailed from 12 different states and 24.0% practiced in an academic setting. Psychiatrist practiced in suburban (56.0%), urban (36.0%), and rural (8.0%) regions.

#### Procedure and Materials

Upon joining the Zoom session, participants indicated consent online. After everyone completed the consent form, the session began, lasting ~30 min. All sessions started with the following prompt: “*To start, we want to get your feedback about a concept we are interested in investigating. Some people think that a patient's expectations of a treatment's effectiveness will influence the actual effectiveness, like a self-fulfilling prophecy*.” This introductory explanation was chosen to provide a common working definition of expectations that was relatable and not overly suggestive. Conversations were then based on the following four questions^1^.

1) Have you heard of the concept of expectancies?
2) How often do you think about patient expectations when interacting with patients?
3) Do you attempt to modify patient expectations? How?
4) Do you think there is anything you do when treating a patient that might influence their expectations, even if that is not your explicit intent?

Demographic data were collected.

#### Setting

Interviews were conducted through videoconferencing from October–November 2020. For the first focus group, MHB served as the primary facilitator while MNR was the secondary facilitator. These roles were reversed for the following six sessions. Both authors asked follow-up questions when relevant. Focus groups were audio-recorded, transcribed by a third party, and anonymized.

#### Analysis

After the first two focus groups, authors reviewed transcripts, which informed which focus group questions were prioritized in the following discussions. The authors (MNR and MHB) conducted thematic coding with the following process: after creating research questions (Supplementary Table S1), they separately reviewed focus group transcripts to determine codes and subcodes, then met and agreed upon a revised list of codes that categorized recurring ideas. Since authors had no a priori hypotheses, an inductive approach was used with thematic analysis (20). Focus groups were independently coded on *Nvivo*. The authors then reconvened to reach a consensus for all codes. Some examples of codes (and subcodes) included office layout (as therapeutic tools, color, credentials, furniture), relationship between expectations and outcomes (how that changes over time), and patient empowerment (goal-setting). Codes were not mutually exclusive and were done primarily at the surface level. MNR reviewed codes, identified central themes, and discussed with MHB.

### Quantitative Study

#### Overview

The purpose of the quantitative study was to further examine topics from the qualitative findings. Specifically, when the focus group study ended MNR and MHB informally reviewed the study transcripts. The authors discussed their perception of what information was captured by this qualitative study, and how it fit with prior research on the placebo effect in psychiatry. MNR and MHB then drafted a series of questions that aligned with the topics from the focus group. Questions were primarily created by the authors, though some were also based on prior studies.

#### Recruitment

Using the same listing as Study 1, the remaining 5,843 psychiatrists who were not recruited for the qualitative study were sent up to three emails from December 2020–January 2021 inviting them to participate in a 9-min survey on “the various factors that impact a treatment's effectiveness.” Everyone who responded verified they were a trained psychiatrist. There were no other exclusion criteria. No compensation was offered.

#### Participants

Participants were an average of 62.72 (*SD* = 13.15) years old, and 33.8% were female. Fifty-four percent, 39.0%, and 7.1% practiced in urban, suburban, and rural settings, respectively; 45.5% primarily saw patients in private practice, while 22.5%, 5.1%, and 0.6% primarily saw patients in a hospital, Veteran Affairs, and retirement/nursing home, respectively (26.3% indicated “other”).

#### Procedure and Materials

Participants indicated informed consent before beginning the survey. In total, 436 people opened the survey, and 346 participated. The study was approved by the Brown University IRB. We assessed expectations with five items, hope with two, placebo effect with three, and aesthetic features with four (Supplementary Table S2).

#### Analysis

Data were analyzed primarily with descriptive statistics. Inferential statistics were calculated using non-parametric tests (e.g., Spearman rho or Wilcoxon Sign Rank Test) since data are ordinal. Analyses were conducted using SPSS version 25 or 26.

## Results

### Overview

Four themes were identified from the focus groups: (1) Expectations, (2) Hope, (3) Placebo Effect, and (4) Aesthetic Features. These themes informed the survey. Below we summarize findings related to each theme from both the qualitative and quantitative studies.

### Treatment Expectations

#### Qualitative Study

Many psychiatrists reported that they were “always” thinking about patient expectations of treatment outcome (Quotation 1). Nearly all participants suggested patient expectations could at least moderately influence outcomes (Quotation 2). Psychiatrists in the focus groups generally believed that either highly inflated or deflated expectations were detrimental to recovery (Quotation 3). Nonetheless, rather than thinking in terms of “raising” or “lowering” expectations, participants usually saw their role as arming patients with truthful information (Quotation 4). This transparency, some explained, could be empowering. In addition, they told patients that medication alone would be insufficient, and patients needed to play an active role in recovery (Quotation 5). Finally, participants in nearly all focus groups organically discussed the importance of the patient-physician alliance in influencing either expectations or outcomes (Quotation 6).

1) “It's always in the back of my mind: about wondering what their thoughts are, about whether they're expecting it to work, and listening for any signals that the patient may be disappointed, excited, whatever.” [Focus Group (FG) 1]
2) “[Patients'] expectations definitely affect outcome, plays a huge role.” (FG 6)
3) “I think people, in general, coming to psychiatry have low expectations, [laughter] so that's a little bit easier to deal with. When people have high expectations, or when they're referred by a friend, and, ‘Oh, you're the greatest. You're wonderful,' that's when I get a little freaked out.” (FG 2)
4) “I find that people are happiest, ultimately, with their medication treatment if they ha[ve] a good understanding going in.” (FG 6)
5) “The idea that this medication is somehow going to completely obliviate any kind of feeling of pain or distress. All it's going to do is bring you back to—within normal limits… if your life sucks, you're gonna feel like shit.” (FG 7)
6) “One of the most important predictors is connection and your faith in the person's capacity to get better and their belief in you believing in them.” (FG 6)

#### Quantitative Study

Nearly all participants (92.2%) thought about patient expectations “most of the time” or “always or almost always.” Participants tried to both raise and lower patients' expectations about medication effectiveness, although they were more likely to raise vs. lower expectations, Wilcoxon *Z* = −8.00, *p* < 0.001 (Table 1). Concerning medication presentation (items 4 and 5 on Supplementary Table S2), participants were split approximately equally between “exactly as optimistic/pessimistic as the most likely outcome” and “slightly more optimistic than the most likely outcome.” Scores regarding what prognosis leads to the best outcome were higher than scores regarding what prognosis participants actually provide, Wilcoxon *Z* = −3.47, *p* = 0.001^2^ (Table 2).

**Table 1:** Attitudes toward patient expectations.

	**Think about patient expectations** **% (*n*)**	**Try to raise patient expectations** **% (*n*)**	**Try to lower patient expectations** **% (*n*)**
Never/almost never	0 (0.0)	9.0 (31)	23.0 (79)
Sometimes	4.0 (14)	39.2 (135)	55.2 (190)
About half of time	3.8 (13)	15.1 (52)	11.3 (39)
Most of time	33.8 (117)	27.6 (95)	7.3 (25)
Always/almost always	58.4 (202)	9.0 (31)	3.2 (11)

*Responses to items 1, 2, and 3 (from Supplementary Table S2) are displayed from right to left*.

**Table 2:** Prognosis attitudes and behavior.

	**Most effective prognosis** **% (*n*)**	**Personal prognosis** **% (*n*)**
Significantly more pessimistic than what you think is the most likely outcome	0.6 (2)	0.0
Slightly more pessimistic than what you think is the most likely outcome	3.0 (10)	3.2 (11)
Exactly as optimistic/pessimistic as the most likely outcome	40.8 (138)	49.3 (170)
Slightly more optimistic than what you think is the most likely outcome	43.8 (148)	44.3 (153)
Significantly more optimistic than what you think is the most likely outcome	10.1 (34)	3.2 (11)
Medication presentation is mostly irrelevant	1.8 (6)	NA; Not response option

*Left column displays answers to item 4 and right column to item 5 (from Supplementary Table S2)*.

### Hope

#### Qualitative Study

Participants spoke about hope as distinct from but related to expectations. They emphasized the importance of being realistic yet still hopeful. Psychiatrists also mentioned “holding hope” for patients as an aspect of their role. They attempted to support and empower patients by being hopeful their situation could improve, even when patients felt demoralized.

“Sometimes it's just a matter of scanning the environment with them and pointing out, help is on the way and it's coming from this direction.” (FG 3)

“If I'm too realistic and I just dash their hopes of getting better quickly enough, then that can also be counter-therapeutic. It is that fine balance that I feel like I have to walk, being realistic but also hopeful.” (FG 4)

“We have to hold the hope for the patient who's not capable of holding that hope themselves.” (FG 5)

#### Quantitative Study

More than 95% of participants indicated that both patient hope and psychiatrist hope were at least moderately important for patient outcomes (Table 3). Patient and psychiatrist hope were significantly correlated: rho = 0.497, *p* < 0.001. The difference between the perceived importance of patient hope and psychiatrist hope was not significantly different, Wilcoxon *Z* = −0.534, *p* = 0.594.

**Table 3:** Importance of patient and psychiatrist hope.

	**Importance of patient hope % (*n*)**	**Importance of psychiatrist hope % (*n*)**
Not at all important	0.0 (0)	0.3 (1)
Slightly important	2.4 (8)	3.3 (11)
Moderately important	20.4 (69)	16.0 (54)
Very important	44.4 (150)	52.1 (176)
Extremely important	32.8 (111)	28.4 (96)

*Responses to items 6 and 7 (from Supplementary Table S2) are displayed in the right and left columns, respectively*.

### Opinion of Placebo Effect

#### Qualitative Study

The placebo effect often naturally emerged as a topic of conversation when interviewers mentioned expectations. Participants were relatively familiar with literature about the placebo effect in psychiatry. They discussed prescribing impure placebos, subtherapeutic doses of medication, and ritualizing medication intake to enhance the placebo effect. A few described the goal as triggering an internal healing process. Some emphasized that while the placebo effect is important, this evidence should not be used to minimize the importance of other aspects of treatment (e.g., active medication, talk therapy).

“Oh, but we prescribe lots of placebos. We just don't know they are.” (FG 3)

“[When we talk about placebos], we're talking about ways that we are mobilizing the healing capacity inside a person and trying to unleash it.” (FG 7)

“I ask them [patients] to take the medication, put it in the palm of their hand… to close their eyes, and to take anywhere from a few seconds to a few minutes to visualize the experience of themselves, that they desire this medication to help them to move into.” (FG 7)

“I certainly prescribe medicines at a dose—like a low dose of an SSRI, which… aren't much good as an antidepressant, and the patients get undepressed. I won't fight with it, but I don't believe it.” (FG 3)

“I don't think everything's the placebo effect, but I think the placebo effect's very powerful.” (FG 5)

#### Quantitative Study

In total, 65.7% of participants indicated that they, at least on some occasions, prescribe a medication that is not significantly more effective than a placebo. In particular, responses were as follows: 34.3% said “never,” 44.1% “infrequently,” 20.4% “sometimes,” and 1.2% “often.” The reasons for doing this are displayed in Supplementary Table S3. Participants estimated that 68.0% (*SD* = 16.0) of an SSRI effectiveness is due to the chemical properties of the pill and 30.9% (*SD* = 15.2) is a result of the placebo effect; 82.1% indicated that the chemical properties of an SSRI are more powerful than the placebo effect, while 6.4% indicated the opposite, and 11.5% gave equal values.

### Aesthetic Features

#### Qualitative Study

Physicians indicated that they actively considered the physical environment where they practice. Many thought their dress could influence patient expectations, although one shared an anecdote suggesting the contrary. Even more participants discussed how they designed their offices to be welcoming to patients. Design features ranged from giving the patient easy access to the exit to inviting a sense of humor with a pillow that says, “If it's not one thing, it's your mother.” The psychiatrists tried to convey their treatment style through the types of artwork on their walls. Some displayed their credentials while others favored a more informal, living-room-like setting. A few worked in a hospital and lacked control over their office layout.

“I think the setting does have an effect on putting the patient in a certain mindset, and it sets sort of a tone to the room.” (FG 1)

“I two years ago dyed my hair multicolor, rainbow… I will tell you the patients connected with me much more…because they saw it as relatable… To them that was just like, ‘This person's more down to Earth and can communicate with me.” (FG 4)

“The patients feel dramatically different in the first appointment [with me after] having come from a mental health clinic… They really feel valued even though probably the psychiatrist at the mental health clinic was treating them just as nicely because [now] they're in this nice setting.” (FG 1)

“I do like to have… one disturbing piece of art [laughter] that says, ‘You know what? I'm not worried about a mess. I'm not worried about things where there's layers and layers and layers, and the more you look at it, the more you see.' Patients are often disturbed, but it's like a—to me, it's a metaphor… to let them know, I'm okay with whatever ugliness is going to be brought in here.” (FG 7)

#### Quantitative Study

Table 4 reports the extent to which participants believed their dress and office design influence patient expectations and outcomes. The perceived importance of aesthetic features all were highly inter-related: Spearman's rhos ranged from 0.517–0.727, *p*s < 0.001 (Supplementary Table S4). Participants indicated that both dress and office design impacted patient expectations more than patient outcome: Wilcoxon *Z* = −9.5, *p* < 0.001, and Wilcoxon *Z* = −8.8, *p* < 0.001, respectively. Participants viewed dress as more influential than office design: Wilcoxon *Z* = −4.2, *p* < 0.001, and Wilcoxon *Z* = −3.2, *p* < 0.001, for expectations and outcomes, respectively.

**Table 4:** Influence of dress and office design on patient expectations and outcomes.

	**Dress influence**		**Office design influence**	
	**Treatment expectations % (*n*)**	**Outcomes % (*n*)**	**Treatment expectations % (*n*)**	**Outcomes % (*n*)**
Not at all	6.2 (21)	20.8 (67)	8.0 (27)	19.4 (63)
Slightly	16.5 (56)	20.5 (66)	19.5 (66)	29.0 (94)
Somewhat	35.4 (120)	30.4 (98)	40.5 (137)	30.2 (98)
Moderately	26.5 (90)	19.6 (63)	23.4 (79)	16.4 (53)
Significantly	15.3 (52)	8.7 (28)	8.6 (29)	4.9 (16)

*The degree to which participants think that dress (left panel) and office design (right panel) influence treatment expectations and patient outcomes (items 11, 12, 13, and 14 from Supplementary Table S2) are displayed*.

## Discussion

There have long been indications that some psychiatric illnesses are responsive to the placebo effect (21), which raises the possibility that patient expectations play a role in recovery. However, despite the increasing interest in understanding non-specific factors in psychiatric treatment [e.g., (15, 22)], no prior studies, to the authors' knowledge, have examined how psychiatrists think about and modify patient expectations. This is a noteworthy gap given that 92.2% of psychiatrists in our quantitative survey indicated they are “always” or “almost always” thinking about patient expectations when interacting with patients.

In the present study, survey responses revealed that psychiatrists frequently raise patient expectations about medications' effectiveness, consistent with research on the powerful role of the placebo effect in antidepressant efficacy (23, 24). Nearly all prior research has indicated that higher expectations promote better outcomes (25) [though see (26)]. However, psychiatrists indicated they frequently *lower* expectations as well; some participants suggested that unreasonably high expectations may be detrimental to recovery. The effect of lowering overly high expectations has not yet been examined in detail among a clinical population [see (27) for an experiment with healthy volunteers]. More broadly, focus groups revealed nuanced ways psychiatrists manage expectations that do not fall neatly into the categories of raising and lowering, such as empowering patients with information, building a strong patient-physician alliance, and encouraging patients to play an active role in their treatment.

One novel feature of the present study is that we explicitly asked psychiatrists about hope in addition to expectations. Prior research in the field of placebo studies rarely focuses on hope, though in two notable exceptions, patients who improved on placebos often said they did not *expect* to improve but rather *hoped* to get better (28, 29). Our study was consistent with the idea that hope and expectations are viewed differently. In particular, hope is more affective and relates to preference, while expectations are more cognitive and relate to perceived probability (30, 31). Interestingly, there was no significant difference between the perceived importance of *patient* hopefulness and *psychiatrist* hopefulness. This survey result complements the focus group concept that psychiatrists could be a support system by “holding hope” for the patient.

On average, participants estimated that 31.0% of SSRI efficacy was due to the placebo effect while 68.0% was due to the drug effect. These are roughly the inverse of academic estimates (11, 32). Our finding is consistent with previous studies in which psychiatrists estimate the placebo effect to be around 26% (19) and 40% (16). This suggests that even though psychiatrists are well-versed on the placebo effect, they still underestimate its contribution to the drug response, at least for SSRIs. In fact, in our survey, only 6.1% of participants thought the placebo effect played a larger role than the drug effect in SSRI efficacy.

### Ethical Implications

This study poses ethical considerations that should be further explored. Although participants on average believed presenting a prognosis with a medication *slightly more optimistically* than the expected outcome would lead to the *best results, in practice*, they reported presenting medications *more realistically*. This difference was significant and highlights a tension between maximizing patient benefit (e.g., setting higher expectations) and respect for patient autonomy (e.g., transparency). The question emerges, is it ethical to enhance expectations (and thereby, presumably, effectiveness) at the cost of some transparency? It is important to consider potential unintended consequences of both strategies. Historical and ongoing (often implicit) medical racial discrimination (33–35) may also be the cause of higher rates of medical distrust among Black Americans (36). What impact would limiting transparency have on people's trust in the healthcare system, especially among individuals in marginalized communities? Conversely, if a medication's efficacy can be maximized, what are the implications of neglecting to raise expectations and improve treatment?

Miller and Colloca (37) have argued that such a trade-off is not necessary; there are ways to boost expectations that do not sacrifice transparency. For example, one study demonstrated that telling patients the percent of study participants who had *not* experienced side effects led to a lower occurrence of side effects than telling them the percent of patients who *had* experienced side effects (38). Further, equalizing the clinical use of the placebo response [e.g., good bedside manner, the process of a thorough psychiatric evaluation, or a warm demeanor; see (39)] could *decrease* health disparities (40).

### Strengths and Limitations

The greatest strength of this study is the mixed methods approach. The focus groups provided texture and depth, while the survey allowed for greater ecological validity. Another strength is that it examines the opinions of psychiatrists across the U.S., rather than at a single institution. Finally, in 2015, 60% of psychiatrists were over age 55 (41), so the mean age of survey participants, 62.72, is likely representative of the general psychiatrist population. However, the recruitment email pool, which roughly mirrored the population of US psychiatrists, was 42% female, so females are somewhat underrepresented in our survey sample (34% female).

One limitation is that focus group participants were told in recruitment emails that the study was designed to investigate “how psychiatrists utilize expectations” (though recruitment emails were vaguer for the survey study). This was necessary for participant recruitment but also introduces selection bias. Participation rates were low, at 0.5% (*n* = 26) and 5.9% (*n* = 346) for the focus group and survey studies, respectively. Demand characteristics introduce another source of bias, as participants may have wanted to please investigators by emphasizing their belief in the role of placebos in treatment. Because the physician sample was limited to the US, findings do not necessarily reflect opinions of psychiatrists in other countries.

Finally, future studies should seek to better differentiate between subspecialties and conditions when asking physicians to quantify the role of non-specific factors.

### Future Directions

Future research should examine *patient* attitudes regarding the tension between transparency and positive expectations. How do patients, across diverse backgrounds (including those with comorbid chronic conditions), want their doctors to maximize the benefit and minimize side-effects of medication while respecting their autonomy? Researchers could also test whether the role of patient expectations differs according to level of chronicity of a condition. One could hypothesize that hope and/or expectations may play a different role in recovery the longer the patient has had their condition. Additionally, because so many psychiatrists indicated that they frequently lower patient expectations, it would be worthwhile to examine if extremely high expectations are iatrogenic. Psychiatrists in the focus groups shared the ways they organically modify patient expectations (e.g., office furniture, clothing, ritualizing medication intake, etc). Future randomized trials could be conducted to examine the effects of these strategies, as well as potential moderators (e.g., demographic characteristics, personality traits, diagnosis, medication type).

## Conclusion

Psychiatrists think extensively about non-specific factors. They modulate expectations in complex ways and tend to believe that non-specific factors play a notable role in treatment outcomes. Still, perspectives are mixed as to the importance of and best ways to harness expectations, hope, the placebo effect, and aesthetic features. Future research should investigate how non-specific factors such as expectations can be maximized to effectively and ethically improve patient outcomes.

## Data Availability Statement

The raw data supporting the conclusions of this article will be made available by the authors, without undue reservation.

## Ethics Statement

The studies involving human participants were reviewed and approved by Brown University IRB. The participants provided their written informed consent to participate in this study.

## Author Contributions

MNR and MHB designed studies and designed the survey. MNR led recruitment with help from MHB. MNR led qualitative interviews and analysis. MHB led quantitative analysis. Manuscript was written in collaboration. All authors contributed to the article and approved the submitted version.

## Funding

MNR was supported by a Karen T. Romer Undergraduate Research and Training Award and MHB was supported by the National Institute of Health grant (K01DA048087).

## Conflict of Interest

The authors declare that the research was conducted in the absence of any commercial or financial relationships that could be construed as a potential conflict of interest.

## Publisher's Note

All claims expressed in this article are solely those of the authors and do not necessarily represent those of their affiliated organizations, or those of the publisher, the editors and the reviewers. Any product that may be evaluated in this article, or claim that may be made by its manufacturer, is not guaranteed or endorsed by the publisher.

## Footnotes

^1^Two other questions are available upon request from authors. They were removed due to time constraints and re-evaluated relevance to research aims. Follow-up prompts are also available upon request.

^2^For this test, we excluded participants who said that prognosis was mostly irrelevant to outcome.
