# Supplementary material for: The Importance of Patient Expectations: A Mixed-Methods Study of U.S. Psychiatrists
Source: Front Psychiatry. 2021 Dec 3;12:781494. doi: 10.3389/fpsyt.2021.781494 (PMC8678457; doi:10.3389/fpsyt.2021.781494)
Supplement: Supplementary file 1 [file Data_Sheet_1.docx]

| Table S1  *Research Questions* |
| --- |
| 1) What are subconscious and conscious ways psychiatrists influence expectations? |
| 2) Do psychiatrists think expectations play a role in influencing outcomes? What role? |
| 3) To what extent do psychiatrists think about expectations? What terminology do they use around expectations? |
| 4) What is the relationship between expectations, hope and honesty?  5) What do psychiatrists think about OLP? |

*Note*: Item 5 was an a priori research question but we did not focus on it due to time constraints.

Table S2

*Survey Items*

| **Item #** | **Item text** | **Response Options** |
| --- | --- | --- |
| **Expectations** | | |
| 1 | How often do you think about patient expectations when interacting with a patient? | 1=never or almost never to 5=always or almost always |
| 2 | When prescribing a medication, how often do you try to raise your patient’s expectation about the medication’s effectiveness? | 1=never or almost never to 5=always or almost always |
| 3 | When prescribing a medication, how often do you try to lower your patient’s expectation about the medication’s effectiveness? | 1=never or almost never to 5=always or almost always |
| 4 | Which of the following presentations of a patient’s prognosis with a new medication typically leads to the best outcome? (assume compliance is the same regardless of presentation) | 1=significantly more pessimistic than what you think is the most likely outcome to 5=significantly more optimistic than what you think is the most likely outcome  6=None of the above; the way a medication is presented is mostly irrelevant to outcome. |
| 5 | Which of the following is closest to the way you tend to present a new medication? | 1=significantly more pessimistic than what you think is the most likely outcome to 5=significantly more optimistic than what you think is the most likely outcome |
| **Hope** | | |
| 6 | How important is it to patient outcomes that the patient feels hopeful? | 1=not at all important to 5=extremely important |
| 7 | How important is it to patient outcomes that the psychiatrist feels hopeful for the patient? | 1=not at all important to 5=extremely important |
| **Placebo Effect** | | |
| 8 | How often do you prescribe a medication that has not been shown to be significantly more effective than a placebo? | 1=never to 4=often |
| 9 | For those who did not answer “never” to 8: To what extent do each of the following play a role in your decision to do so? | 1=not at all to 5=very much  (Six potential reasons were provided and can be found in Table S3) |
| 10 | On average, what percentage of an SSRI’s effectiveness is due to each of the following: 1) Chemical properties of the pill; 2) Placebo effect. | Participants were asked to fill in percentages for each that added to 100%. |
| **Aesthetic Features** | | |
| 11 | Do you think the way you dress influences patient expectations about how effective a treatment will be? | 1=not at all to 5=significantly |
| 12 | Do you think the way you dress influences patient outcomes? | 1=not at all to 5=significantly |
| 13 | Do you think the way you design your office influences patient expectations about how effective a treatment will be? | 1=not at all to 5=significantly |
| 14 | Do you think the way you design your office influences patient outcomes? | 1=not at all to 5=significantly |
| *Note:* Only survey items reported in the present study are listed. Some additional questions were also asked. | | |

Table S3

*Reason for prescribing medication not known to be significantly more effective than placebo*

|  | **The action of taking pill can help**  **%** | **Appease patient who wants medication**  **%** | **Active ingredient could help**  **%** | **Harness placebo effect**  **%** | **Few side effects**  **%** | **No better alternative**  **%** |
| --- | --- | --- | --- | --- | --- | --- |
| **Not at all** | 16.0 | 27.1 | 6.0 | 20.1 | 4.2 | 3.7 |
| **Slightly** | 26.0 | 35.8 | 14.3 | 25.1 | 13.5 | 11.1 |
| **Somewhat** | 31.1 | 23.9 | 27.6 | 29.7 | 20.0 | 27.6 |
| **Moderately** | 17.4 | 9.2 | 36.4 | 16.4 | 31.6 | 31.8 |
| **Very much** | 9.6 | 4.1 | 15.7 | 8.7 | 30.7 | 25.8 |

*Note:* Frequencies are displayed. Responses only provided for participants who did not indicate they “never” prescribe a medication that has not been shown to be significantly more effective than a placebo.

Table S4

*Relation between perceived importance of aesthetic features*

|  | **Dress/Expectations** | **Dress/Outcomes** | **Office/Expectations** | **Office/Outcomes** |
| --- | --- | --- | --- | --- |
| **Dress/Expectations** |  |  |  |  |
| **Dress/Outcomes** | .727*** |  |  |  |
| **Office/Expectations** | .593*** | .544*** |  |  |
| **Office/Outcomes** | .517*** | .678*** | .717*** |  |

*Notes*: Spearman’s rho are displayed. Study variables are: the extent to which psychiatrist dress is perceived to influence patient expectations (column/row 2) and outcomes (column/row 3), and the extent to which office layout is perceived to influence patient expectations (column/row 4), and outcomes (column/row 5). *** p<.001, two-tailed
